# Engines of alternative objectivity: Re-articulating the nature and value of participatory mental health organisations with the Hearing Voices Movement and Stepping Out Theatre Company

**DOI:** 10.1177/1363459315590246

**Published:** 2015-06-25

**Authors:** Claire Blencowe, Julian Brigstocke, Tehseen Noorani

**Affiliations:** University of Warwick, UK; Cardiff University, UK; Johns Hopkins University, USA

**Keywords:** Alienation, authority, Georg Simmel, Hearing Voices Movement, mental health, objectivity, participation, performance, Stepping Out

## Abstract

Through two case studies, the Hearing Voices Movement and Stepping Out Theatre Company, we demonstrate how successful participatory organisations can be seen as ‘engines of alternative objectivity’ rather than as the subjective other to objective, biomedical science. With the term ‘alternative objectivity’, we point to collectivisations of experience that are different to biomedical science but are nonetheless forms of objectivity. Taking inspiration from feminist theory, science studies and sociology of culture, we argue that participatory mental health organisations generate their own forms of objectivity through novel modes of collectivising experience. The Hearing Voices Movement cultivates an ‘activist science’ that generates an alternative objective knowledge through a commitment to experimentation, controlling, testing, recording and sharing experience. Stepping Out distinguishes itself from drama therapy by cultivating an alternative objective culture through its embrace of high production values, material culture, aesthetic standards. A crucial aspect of participatory practice is overcoming alienation, enabling people to get outside of themselves, encounter material worlds and join forces with others.

## Introduction

After decades of campaigning and felicitous shifts in state structures, there is now widespread acceptance that service user participation is an important component of mental health service delivery and support (Department of Health, 1999, 2011; England et al., 2014; Lewis, 2009; Martin, 2008). However, this importance remains under-theorised and is often misunderstood (Gibson et al., 2012; Nathan et al., 2014). Participation tends to be framed either in terms of ‘giving voice’ or of enabling effective consumer choice and feedback (Beresford, 2002; Mullen, Hughes & Vincent-Jones (2011).

A key difficulty with the usual understandings of participatory practices in mental health is that conventional dichotomies about what is and is not ‘science’ or ‘objectivity’ remain unchallenged, such that participatory practices and user perspectives are always assumed to be the *other* of both science and objectivity. In particular, the biomedical model (Deacon, 2013; Engel, 1977) is often assumed to have a monopoly on scientific objectivity, while participatory practices are characterised as ways of expressing so many individual subjective perspectives – even by advocates of participation (Mattingly, 2005; Moynihan and Cassels, 2005). The retention of the conventional dichotomy is problematic because it radically underplays the importance of committed ethos, knowledge sharing, collectivisation of experience and engaging with material reality – which are facets of *objectivity* – to participatory practices themselves. We focus on these objectivity-oriented aspects of successful participatory practice and do so in order to emphasise the role of collaboration in that success (see also Armstrong and Murphy, 2012; Horsfall, 1998; Raby, 2012). Drawing on science studies and the sociology of culture, as well as traditions of self-help and peer support, we argue that the value of participatory practice should also be understood in terms of overcoming alienation: an overcoming that is constituted precisely through the generation of ‘objectivity’, which is to say, the collectivisation of experience. As such, participatory organisations should not be seen as the subjective ‘other’ to the objectivity of science, but as organisations acting as ‘engines of alternative objectivity’. With the term ‘alternative objectivity’ we point to modes of sharing and concretising experience that are different to biomedical science, but which nonetheless *are* forms of objectivity. The term is intended to echo Gibson-Graham’s (2006) work on ‘alternative economies’.

In this article, first we look to the political tradition of participatory democracy to draw out the relationship between participation and overcoming alienation – a relationship that is generally overlooked in the mental health literature. We conceptualise the relationship between alienation and objectivity with reference to Georg Simmel’s sociology. Second, we set out a pluralist approach to objectivity. We then consider two successful participatory organisations and the ways in which they generate objectivity: the Hearing Voices Movement and Stepping Out Theatre Company. The Hearing Voices Movement has cultivated what we refer to as an ‘activist science’ by incorporating an ethos that generates objectivity through commitment to experimenting with, controlling, recording and sharing experience. Through these commitments, voice hearers attempt to come into a richer, augmented, relationship with reality. In contrast to the usual style and priorities of community theatre or theatre-as-therapy, Stepping Out Theatre Company has actively cultivated and participated in the ‘objective culture’ of the theatre world through its embrace of high production values, aesthetic standards and critical review processes. This augments the capacity of the group to enable members to overcome alienation and participate in objective experience. We conclude that recognition of the diverse forms of objectivity and authority that emerge through modes of collectivisation is vital for grasping the importance of participatory practice in mental health today.

## Methodology

The article draws on research conducted by one author with the Hearing Voices Movement between 2007 and 2011 (Noorani, 2013). Data cited in this article include quotes from 12 semi-structured interviews with members of the Hearing Voices Movement at local level (with a group facilitator and with long-term group members) and with a key member of the national UK Hearing Voices Network. The research data were gathered iteratively from 2009 to 2011, using subsequent encounters with participants to gain a better grasp of the themes that had emerged through the interviews. As a result, some participants were formally interviewed more than once. The author has also spent 8 years as a volunteer ‘ally’ of the steering group of a hearing voices self-help network. Other data informing the analysis include notes from attending self-help group meetings (by invitation), including steering group meetings with a local hearing voices group. In addition, the author had informal discussions with local service user and survivor activists over the period of data gathering, from autumn 2008 until early spring 2011. Alongside these data, we have analysed Hearing Voices Movement self-help publicity materials such as group leaflets, Internet sites, including web-based fora discussing the common problems related to experiences of distress, and campaigning, lobbying and advocacy groups and initiatives related to the self-help organisations.

The article also draws on two interviews conducted by the authors with key representatives of Stepping Out Theatre Company, as well as observations of the company’s performances and analysis of newspaper theatre reviews of the Company. The authors also conducted a review of participatory theory and developed and tested the ideas for the article through conversations with a group of 12 academic-practitioners of participatory pedagogy, democracy and science in a residential retreat in 2012 (see also Noorani, Blencowe & Brigstocke, 2013).

## Participation and alienation: the importance of objectivity

Participation in mental healthcare is usually interpreted along consumerist or democratic frameworks, either as ‘improving services and choices’ or ‘giving voice to the voiceless’ (Beresford, 2002). The concept of alienation is rarely found in analyses of mental illness and health (for notable exceptions, see Evans, 1978; Laing, 1990; Turnbull, 1997). Alienation is, however, a central concern of the political tradition of participatory democracy and pedagogy, especially the Latin American tradition associated with Paulo Freire, Liberation Theology, the Zapatistas and the Porto Alegre experiments in participatory budgeting and democracy (Pearce, 2010). A key concern of that tradition is to overcome situations of alienation that are wrought by dispossession, poverty, authoritarianism and globalisation. In that tradition, overcoming alienation is understood as synonymous with the attainment of dignity (Holloway, 1996). We argue that work towards overcoming alienation and attaining dignity is in fact also central to the success of participatory mental health organisations. Grasping these processes theoretically requires a shift in the way that the relationship between participatory practices and objectivity is usually conceptualised in mental health.^1^ What enables people to overcome alienation is collectivising and objectifying experiences, not giving voice to subjective experience or mobilising consumer choice.

Objective knowledge and culture are central to overcoming alienation (see Blencowe, 2013b). As Jason Read (2010) argues, alienation is not about the loss of subjectivity for individuals but is rather about ‘the loss of objectivity for the subject’: ‘[A]lienation is a separation from the condition of the production of subjectivity; it is not a loss of what is most unique and personal but a loss of connection to what is most generic and shared’ (p. 124). The work of early 20th century social theorist Georg Simmel is useful for conceptualising the relationship between objectivity and alienation (Frisby and Featherstone, 1997). Simmel distinguishes between objective and subjective culture. ‘Objectivity’ describes the forms through which people share experiences, including objects (such as texts, works of art, buildings or tools), standards, established practices and styles. ‘Subjectivity’, in contrast, denotes the individual experience of life: the desire, dissatisfaction, striving and enjoyment that create endless restlessness and reinvention – seeking meaning, breaking forms, working towards new ones. Cultural development takes place in the movement back and forth between objectivity and subjectivity. Objective culture constitutes the grounds of collective life. It enables the pooling of resources and the collectivisation of experiences, empowering people to become more than individual and more than opinion. It offers means of aggregation: media for the connection of capacities.

In terms of Simmel’s account of culture as the movement between the subjective and the objective, alienation results from the separation of the subjective from the objective. Here, the flux through which people move outside of themselves to enter into the shared, objective, world is interrupted, creating a solipsistic subjective condition. Often this happens because projects of objective culture become too complex, vast or rigid to be understood, appropriated and transformed by subjective life. Simmel calls this condition ‘over-objectification’ – when an objective form can no longer be appropriated into the understandings and creative actions of subjects – and it is an apt description of our contemporary relationship to the highly specialised, vast and heavily invested objective cultures of biomedical practice and knowledge.

Simmel suggests that the conditions of metropolitan, capitalist (and we should add *biopolitical*) society tend towards widespread over-objectification, such that we come to feel ‘estranged from form itself’ (Frisby and Featherstone, 1997, pp. 55–101, 174–186). Feeling that objective form is an affront to life, rather than a part of it, we feel ‘objectified’, and this often leads to a rejection of the claims and tools of objectivity *per se* – a retreat into the subjective. However, as Read (2010) suggests, alienation can only be overcome by appropriating – not rejecting – the objective. To flee the objective and embrace only the subjective realms of individual experience, movement and flow are to remain stuck in the solipsistic condition. Thus, while the objectification of people through the biomedical model has rightly been a target of pro-minority mental health politics, the development of creative subjective capacity, in our analysis, is not possible without creating and entering into the objective. Dignity can only be achieved in association. As such, a pro-minority politics of participatory democracy is about subjects moving outside of themselves to join forces with others, entering into relationships with the world and becoming renewed through such encounters (see Kirwan, 2013). This includes encounters with matter and forces that are not always human and certainly not always the same. Things, objects, ‘materialised’ relations of authority, established distance, technologies – these material components of the world are rightfully the tools, not enemies, of subjective development and empowerment. Overcoming alienation involves appropriating, claiming and using what is objective; it means ordinary people taking up objectivity, capacities of the collective and material world, as a part of their own creative becoming.

## Objectivity is plural: insights from science studies and sociology of culture

Before elaborating on the ways in which participatory organisations generate alternative objectivity, we will reflect briefly on science studies and the sociology of culture in order to firmly establish the idea that the pursuit of objective knowledge is not a singular practice carried out by a particular type of expert but is rather an essentially plural and diverse set of practices (Blencowe, 2013a).

The idea of objective knowledge often appears in discourses as a way of dismissing the experiences of ordinary people as mere subjective opinion, holding no weight against the towering ‘objective’ view of the sanctified experts of science. However, no subject, scientist or otherwise, can claim a total and exclusive access to what is objective, for objectivity is precisely that which is beyond *any* specific perspective – it is beyond or outside of subjectivity. Historians of science Peter Galison and Lorraine Daston (2008) explore the meaning and history of the term ‘objectivity’ in the context of practices of scientific observation. They demonstrate that the term only came to prominence at the end of the 19th century, as the accompanying definitional *other* to the individualistic modern subject. The opposition between the objective and the subjective is, then, relatively recent and is bound up with the historical–sociological phenomenon of modern individualism. They argue that objectivity should not be seen as a particular type of knowledge but rather as an *ethos* – a practical ethics and set of habits through which one expresses and practices commitment to science. Moreover, they show that within modern science, there exist diverse approaches in pursuit of objectivity – from mechanical observation to the art of practicing ‘trained judgement’.

More broadly, Science and Technology Studies has long maintained that objectivity is not a single thing or perspective (Harding, 1991; Latour, 1999). Objective, material, reality is by definition external to our human experience. Objective knowledge is generated through controlled encounters with the world. Therefore, any single knowledge or knower cannot capture an objective reality in its totality – the very fact that it is an objective reality means that it is beyond the total grasp of a given knower. As such, it will always be possible to approach a matter in different ways, generating additional knowledge – sometimes complementing, sometimes overturning, sometimes quite irrelevant to the existing knowledge. Different ways of approaching the same substance or event lead to different understandings, but that does not mean that they are not both objective knowledge. A biologist, a geologist and a physicist all know the same object ‘objectively’, although they know ‘it’ in very different ways. Moreover, we have become increasingly aware that material reality itself is in significant part constituted through practices of paying attention, observation and measurement (Barad, 2007). As feminist philosophy of science has demonstrated, discourses that deny the plural nature of objectivity and posit such a thing as the singular objective or scientific perspective are ideological (not scientific), ignoring the self-evidently diverse and open nature of scientific enquiry and undermining the quality of such inquiry (Haraway, 1988; Harding, 1991).

The following two sections introduce our two case studies – the Hearing Voices Movement and Stepping Out Theatre Company. The former is an interconnected, heterogenous global movement, while the latter is a locally-based organisation and legally incorporated charity. Nevertheless, we consider it productive to view them together, as different but complementary examples of combatting alienation through the pursuit of objectivity. By bringing the Hearing Voices Movement and Stepping Out into proximity with the challenges of participation, we hope to question the presumption that what is valued under the rubric of ‘participation’ in mental health should be limited to the participation of individuals in an already established and external statutory service provision or formal research practice. The risks and negative effects upon health and well-being of individual service user participation and involvement have been noted elsewhere (e.g. Snow, 2002; Stamou, 2010). Here we suggest a new framing of the issues in terms of collective empowerment through investments in forms of objectivity.

## The Hearing Voices Movement and pursuit of objective knowledge

Since its inception in the chance encounter in 1987 in the Netherlands of a psychiatrist and a patient who resisted the biomedical framing of the voices she heard, the Hearing Voices Movement has grown into a global set of overlapping peer support networks, with hundreds of self-help groups in over 26 countries. The Movement is centred around the sharing of personal stories of voice-hearing in peer-led self-help groups. The overall aim of the movement is to encourage the personal exploration of these and other unusual experiences by ‘working through’ rather than ‘repressing’ such experiences (Intervoice, 2013a). Affiliated umbrella bodies, such as the UK and US National Hearing Voices Networks, provide documentation and guidance for local groups and individuals on how to set up and maintain effective self-help group practices.

The goal of the Hearing Voices Movement is not for members to get rid of their voices but to develop better relationships with voices. A key premise of the movement is that hearing voices is a natural part of the diversity of human experience, and is problematic only if distressing for the voice hearer. Through group meetings, individuals who have similarly unusual experiences come to realise that they are not alone, building empathy, reassurance and hope around shared projects for working upon their experience (Blackman, 2001; Borkman, 1999; Noorani, 2013). Through structured storytelling, group attendees share examples of what does and does not work for them, prompting others to experiment with how they engage with their own voices. This is done within designated ‘safe spaces’, where groundrules shape how participants can interact, allowing the hegemony of biomedical frameworks to be set aside, providing a precondition for group meetings to act as incubators for diverse ideas. At a collective level, novel ways of speaking are made possible by new concepts and metrics for comparing, including experimental techniques for coping with, and transforming, distressing experiences.

To date, the Hearing Voices Movement has largely evaded flows of capital that connect pharmacological research and development across private pharmaceutical and university sectors globally. Much has been written on regulatory capture and the gaming of the publication industry, such as through cherry-picking evidence and ghost-writing (Goldacre, 2012). Within academia, psychiatric research tends to draw upon standard hierarchies of evidence to identify the randomised controlled trial (RCT) as the ‘gold standard’ of research (e.g. Healy, 2012). Yet while methodologically attuned to the biomedical paradigm, RCTs are woefully inadequate for assessing self-help and peer support practices. In addition, academic researchers seeking to partner with the peer support groups have begun from the assumption that the groups are repositories of individual (or even anecdotal) knowledge, in need of authentication through processes of blinded or statistical aggregation (Corstens et al., 2014: 289–290).

In defining itself in opposition to these dominant authorities, the Hearing Voices Movement is easily and often characterised as the *other* to science and objectivity. However, this is not enough to explain its substantial influence and growth. For that, we need to appreciate the *positivity* of the movement in producing an experiential knowledge-base and an objectivity generated through its own experimentation and knowledge aggregation practices. Images and figures, for example, are crucial units of knowledge, by linking diverse meaning-making systems and material bodies in new ways (Haraway, 2007: 4). The Hearing Voices Movement is not simply giving voice to service users but is generating new objective knowledge about the experience and transformation of voice-hearing. It has rejected the way in which voices are seen as meaningless in biomedical discourse, choosing instead to understand voices as ‘messengers’ to be engaged with.

The idea of voices as meaningful – whether as identities or otherwise – conflicts with the traditional biomedical view that voice-hearing is simply an indication of what is ultimately a physical illness in the brain. Within the Hearing Voices Movement, understanding one’s voices – like getting to know a stranger – is an unpredictable and messy process, not easily amenable to assessment via external yardsticks of wellness or progress. Nevertheless, the Hearing Voices Movement literature does distinguish different phases in voice-hearing, arranged in accordance with the end goal of being able to say, ‘I hear voices and I’m happy about it’ (Intervoice, 2013b). For many (though not all), the journey is one of ascertaining the original biographical events at the source of the voice(s) in order to begin the process of gaining control over the voices and changing voice hearers’ relationships to voices, such that they are no longer distressing (Corstens et al., 2014; Dillon, 2010). According to such a trauma-based approach, the need to dissociate from distress creates identities that may have been helpful at a given time, but can be extremely unhelpful if they return, perhaps years later, as voices, acting as old ‘solutions’ to long-gone ‘problems’. As such, collective knowledge in the Hearing Voices Movement includes lists of salient factors to be considered in decoding voices: the time the voices began, their names, the number of them, when they intervene, what triggers them and what they say can all provide clues for ascertaining their ‘identity’ (see Longden et al., 2012; Romme and Escher, 2000). The process of making sense of voices, enacted and encouraged through self-help meetings, is understood through the Hearing Voices Movement as a gradual one that can begin by teasing out distinct voices from the mélange of noises experienced, then decoding the metaphorical content of what they are saying and understanding when, how, why and as whom voices are conveying it. By acknowledging and understanding the stable properties of these voices, voice hearers are better able to predict their activity and engage with them in new ways.

The above examples suggest that the range of techniques and modes of encountering voices that have emerged over the years are concerned less with escaping, rejecting or opposing the experience of voice-hearing than with working with it and on it, deepening the experience and understanding it better (see Coleman and Smith, 1997; Martin, 2006; Smith, 2001). The processes of making sense of voices is understood as a gradual one that enrols a range of techniques and tactics in transforming one’s relationship to one’s voices (Longden et al., 2012; Romme and Escher, 2000). Experiments in questioning voices are deemed successful when voices enter into productive alliances with voice hearers, enabling the latter to engage with their voices differently and with greater insight into their nature.

We suggest that one reason for the Hearing Voices Movement’s success that has not been fully articulated in the research literature is that the Hearing Voices Movement is an ‘engine of alternative objectivity’. Within the movement, objective knowledge is produced through self-experimentation and knowledge sharing. The transformed experiences of individuals constitute the evidence that the knowledge produced through groups ‘works’ in developing deeper and clearer relationships with voices. Experimentation is a way of testing reality, of coming into a richer relationship with it, allowing the possibility of failure and hence the possibility of capturing reality better. As one Hearing Voices Movement member recollects of her own experiences in attending a hearing voices group,

it was very organic, like just sitting in groups with people and … somebody would talk about … this dominant voice that was really oppressing them. And we’d literally say, well alright, well I’ll be that voice [pointing to herself], and I’ll be you [pointing to someone beside her], and you now tell us what happens … we’d do drama, we’d do all sorts of things really. And it would literally be just experimenting with trying to, sort of, help people deal with their voices really. You know, just trying it out. (Interview with Hearing Voices Movement member)

The knowledge emerging from the Hearing Voices Movement gains depth and weight as across hundreds of self-help groups, people are in effect testing ways of understanding and engagement with voices. At the same time, the involvement of members in the production and regulation of collective knowledge raises promises of a new and deeply participatory form of practice.

Also crucial for the creation of objectivity are ‘communities of knowledge’. In the Hearing Voices Movement, this communication process is manifest in storytelling practices in which members share the results of their experimentations with their voices (and/or related phenomena). Storytelling is crucial to how the Hearing Voices Movement groups share examples of what worked and what did not work for particular individuals in specific contexts. The coalescing of knowledge at the collective level around triggers, modes and techniques of engagement and coping strategies all illustrate the production of objective, shared knowledge (see also Blackman, 2001).

It is in this light that we propose the Hearing Voices Movement’s ‘experiential authority’ (Blencowe, 2013a; Brigstocke, 2013a; Dawney, 2013; Noorani, 2013) is predicated upon a collective knowledge of experience at the limits of intelligibility. The movement encourages an engagement with the distress caused by voice-hearing through experimental projects aimed at deepening understandings of oneself and one’s voices (Intervoice, 2013b, 2013c). The authority of long-term members of the movement granted by newcomers mirrors the authority of the movement as a whole granted by those external to it: in both cases, the granting of authority is tied to the depth and breadth of experiential knowledge as evidenced by the fact that these techniques work in changing relationships with voices. By focusing on experiences that are unique to them, voice hearers have access to experience at the limits of comprehension, which can be worked upon in ways that yield knowledge. The slow building of a robust and collective evidence base for coping with voice-hearing and transforming relationships with voices, through methods of self-experimentation and the sharing of stories amongst peers, is a very different process of knowledge production than the dominant statistical ones driving biomedical and neuroscientific paradigms.

While members and allies of the Hearing Voices Movement often view their practices in opposition to ‘science’, as equated with biomedical research and knowledge claims, we are suggesting that the Hearing Voices Movement itself exemplifies the commitment to experimentation and knowledge sharing that is at the heart of the scientific ethos, and that the movement itself produces objective knowledge. That is to say, the Hearing Voices Movement, through experimentation and the collectivisation of experience, does not simply offer an ‘alternative perspective’ to that of the biomedical model. It offers different means for entering material reality: an alternative objective knowledge. Moreover, rather than merely being ‘fodder’ for academic research or an alternative to mainstream mental health service provision, the Hearing Voices Movement occupies a distinct position as an activist science-generating community in its own right – presenting the possibility of participatory, democratic science. It offers an alternative authority predicated upon shared and tested knowledge and clear techniques and tactics for working upon experience.

Of course, we must be cautious in suggesting that the Hearing Voices Movement is *necessarily* empowering or emancipatory. The Hearing Voices Movement approach does not work for everyone, and therein lies a real risk of not hearing people’s suffering once again, by forcing it into a particular explanatory framework. Nevertheless, recognising that the Hearing Voices Movement produces a distinctive knowledge base and authority through experience moves us beyond the blackmail of being forced either to accept or to reject the ‘truth’ of a biomedical framework (Deacon, 2013; Foucault, 2000).

## Stepping Out Theatre Company: objective culture as engagement with aesthetic standards and material culture

Building on recent work on the role of performance in mental health practices (e.g. Williams, 1998) and on the capacity of performance to generate new forms of authority and objectivity (Brigstocke, 2012, 2013, 2014; Millner 2013) we now turn to a second case study to complement our analysis of participatory practice in the Hearing Voices Movement. Stepping Out Theatre Company is a registered charity based in Bristol, UK, where mental health service users have collaborated in putting on theatrical productions since 1997. These productions range from small one-off performances to a large annual show that runs for weeks, often touring the United Kingdom. The aims of the troupe are to relieve the conditions of mental health service users through the medium of theatre, to advance the education of the public about mental health and to advance the education of mental health service users and allies to develop their creative talents (Stepping Out Theatre, 2013). While the Hearing Voices Movement pursues objective knowledge through activist science, Stepping Out bucks the usual trend in community or therapy theatre by stringently pursuing the highest aesthetic standards and quality: participating in objective culture. As we will illustrate below, an engagement with material culture of craft and technology is essential for such a pursuit.

Stepping Out uses theatrical performance to build intense collectivities and to facilitate empowerment by embracing the standards and values of quality performance. Their plays often contain ‘anti-biomedical model’ messages, in more or less explicit ways. More important than such messages, however, is the way in which the group challenges stigma performatively, in the very act of putting on plays where the actors have all used, or currently use, mental health services. The performative aesthetic is more important than the explicit pedagogy (see also Bell, 2011) – the actors are doing things that ‘those people’ are not ‘supposed’ to be able to do. Troupe director Steve Hennessy explains that audiences are regularly impressed when they hear stories of actors who not long ago were unable to get out of bed are now performing in plays:

The plays challenge psychiatry, but they also challenge stigma about mental health problems. Generally, tabloid messages have negative and warped ideas of people with mental health problems, so anything to counter that is a large part of our remit. So having people performing and dancing etc. and having the audience go, oh wow that’s quite good, and wow these people have really worked hard, it really challenges people’s ideas of people with mental health problems. (Interview with Hennessy, 2012)

The ambitions and achievements of Stepping Out can be understood as contributing to the creation and appropriation of objective culture in the service of minority empowerment. This occurs in several ways. First, Stepping Out members are centrally concerned with creating and becoming a collective entity, over and above the subjective lives of individual members. Second, as we have noted, the troupe takes pains to affirm that its work is ‘real theatre’ and to subject its performances to the same processes of aesthetic critique that apply to any other play. Finally, we highlight the role of material culture and technology – photography, lighting, make-up and the stage itself – in achieving these manifestations of objective culture. We will develop these points in turn.

First, it is clear that building a collective entity is crucial to what Stepping Out achieves: ‘Theatre, music and the arts are bonding and collaborative processes’ (Interview, 2012). One academic commentator notes, ‘the feeling of comradeship and collaboration … is at the heart of Stepping Out’s work. Indeed, one member articulated the company as “involvement, companionship, awareness, and a family”’ (Harpin, 2010: 42). The troupe works to create a commons: what Simmel might call a *form* of life. This collective form includes having a reputation for putting on quality performances in shared spaces with professional equipment and intense preparation. Hennessy describes the effect of the big annual performance put on once a year in creating ‘… a community based on a very intense experience that most people take part in, and then lots of other things that keep the energy going until the next [year’s] big show starts up’ (Interview with Hennessy, 2012).

There is a constant movement of influence back and forth between the objective form, which is the troupe or the play, and the subjective vitality of members. The troupe experiments with developing a craft of living in common. Part of this craft has entailed a refusal to become fixated on ‘problems’. Rather, problems are described as often falling away, become obsolete through creative collaboration. For example, Hennessy describes an actor who threatened to derail the whole production when she became very frustrated about the part she was given in a play. Over time, the director and supporting cast were able to carve out a distinctive character for her, and as the momentum of the play grew, she became very happy with the play and her role within it.

This inclusive growth model relies on an experimental, improvisatory approach:

I can have a conversation with someone in the group and it sparks an idea, and something new emerges – a new play, or a new part. Create, combine, stay open and flexible … because you’ve created roles that can fit anything people want to do, you can be genuinely inclusive. (Interview with Hennessy, 2012)

Moreover, after every annual show, the Stepping Out casts feedback on each developmental stage of that year’s play: the preparation period, the performances, memorable moments and spaces for improvement. This represents the members’ chance to exercise an influence over how the project will be run the next year. In turn, the subjectivity of group members is itself perpetually transformed through participation in the collective. Stepping Out’s shared passion in the art form that brings members together allows mental health issues to take a secondary role, which Hall describes as a ‘relief’, after having had their mental health problems be considered their most important – if not defining – characteristic. Interviewees report how new members are lured by a shift from being a service user/patient to an actor whose capacities can be drawn upon and recomposed. As Hennessy quips, ‘… you can come to us and be a mental health service user, or you can come to us and be Dionysus God of Theatre! The choice is yours, but I know what I would choose’ (Interview with Hennessy, 2012).

As a collaborative space and source of affirmation, the troupe validates possibilities of ‘being otherwise’ – creating and augmenting members’ capacities. A crucial aspect of this transformative, augmenting potential is the challenge and hard work involved in putting together a high-quality production, requiring over 3 months of intensive work. Consequently, members have a real ‘sense of ownership’ of the performance and its reception, profoundly affecting their self-esteem (Interview with Hall, 2012).

Stepping Out explicitly rejects a therapeutic evaluation framework. Stepping Out does not see itself as a form of ‘drama therapy’, where the plays are primarily judged in terms of their therapeutic effect. Rather, Stepping Out members insist that their first priority, and day-to-day concerns, are focused upon the *quality* of the plays they put on. Performing an outstanding play is a collective accomplishment. It produces an intensity of experience which exists communally and can be measured both through the bodies of actors and through audience feedback. The flavour of their productions enhances such effects by contrasting vividly with the themes of passivity and pathology that abound in mental health contexts: ‘one can readily perceive how its high-octane contortions of tragedy, gender, and fooling are statements of transgression in a space of fixity and pathology’ (Harpin, 2010: 54).

In contrast with social inclusion agendas, Stepping Out does not use participation as a proxy measure for recovery. Through Stepping Out, mental health service users enter into growth processes – a kind of *becoming-actor*, which demands qualities such as perseverance, engaging with fear, discipline and learning to welcome the unexpected. These capacities help improve mental health, and yet mental health is never the explicit focus:

although the motivation is to improve mental health, the focus is … on putting on a production … What you focus on becomes a massive part of your life, and with mental health, can tend to have the effect of amplifying the effects on you. (Interview with Hall, 2012)

Rather than measures of recovery, aesthetic standards of theatre culture are central. The creation of collective life through theatre is not simply about the emotional closeness and collective experience of the cast. It is also, crucially, about encounters with the external standards of theatre. As Hall explains, ‘The quality of the theatre that comes out at the end is a really important part of the healing process. It does matter how good it is, how much effort you put in, and so on …’ (Interview with Hall, 2012).

The company embraces aesthetic standards by engaging with the established forms of criticism in the theatre industry. Above all, this means courting critics’ reviews and taking them seriously: ‘The way the industry judges us and critics judge us is really important, and if that pushes us more and more into the mainstream, to be seen as artists rather than service users, that’s really positive and important’ (Interview with Hennessy, 2012). Reviews are posted on their website, reflecting the quality of their work. Of the recent play ‘Five Kinds of Silence’, *The Public Reviews* states that ‘The writing is stark and unflinching, but still able to allow for occasional dashes of humour. The characters in the play are people who live amongst us … Even in its title, this play asks questions of us all’. And *Plays International* states,

… director Chris Loveless and a stunning cast have created a totally gripping piece of theatre which, while deeply moving, allows us to engage with compassion and even with hope … This extraordinary production … has pin-drop attention and passionate applause from the full-house first-night audience so if you’re anywhere near London, book while you can.

In addition to the professional reviews, the troupe distributes evaluation forms among the audience at the end of each play. Members analyse the forms to check and deepen their understandings of what worked and what did not, and what can be improved upon in the next play.

The objective aesthetic standards are crucial to empowerment in Stepping Out. As Hall reflects, ‘[t]hat’s part of empowering people; it’s not only giving them the power to do things but also … to know that the effort they put into things does matter’ (Interview with Hall, 2012). Here, dignity and equality are attained not by reducing all to the lowest denominator and saying that quality is not important, that ‘it’s only the taking part that counts’. Instead, equality and dignity are achieved through assuming and demanding that everyone in the company can and should contribute to making genuinely good quality theatre. Hall says, ‘We often notice the audience playing a game of trying to spot the professional actors and that game demonstrates that it is difficult to distinguish them, which shows that everyone is capable and has skills to offer’ (Interview with Hall, 2012).

Finally, we note the importance of material culture and technology in what Stepping Out does. Stepping Out *looks* like ‘real theatre’ (not ‘am-dram’), and this creates a sense of dignity and self-esteem among troupe members:

The production values are crucial … – professional costumes, there’s a big professional make-up team, some people take 30 minutes or 45 minutes to get their make-up ready. That is part of the alchemy and the magic and the ritual of it – the costumes, the make-up, professional lighting and technicians, all of those things that raise the bar and make them realize, ‘I am a professional in a proper theatre space with standards’. (Interview with Hennessy, 2012)

The high-quality publicity materials provide evidence of, and record, the aesthetic standards of production that are achieved:

We have a proper graphic designer who’s working for us … Also we use a professional photographer and video person, so you’ve got a permanent record of the show, people take away really good classy photos of themselves on stage, DVDs for people to show their friends, all these things make people feel it is really special. (Interview with Hennessy, 2012)

Technologies of display – lighting, make-up, stage – augment performances and help performers and audiences enter into the play as ‘theatre world’. Technologies of recording – photos, DVDs, published reviews – materialise the transient instance of the performance, constituting the show as so many objects that members can come back to, a reminder of success, intensity and becoming, as well as material to draw bodies, capacities and knowledge towards the next production. Stepping Out does not simply create a space for individuals to come and be included and feel good. It assembles various bodies and forces to enable people to become *more-than* what they are, to step outside of their subjective lives, producing objective cultural *forms* – the theatre troupe, the play, professional aesthetic standards and the audience’s knowledge of mental health.

## Conclusion – overcoming alienation through alternative objectivity

We have argued that the participatory practices of Hearing Voices Movement and Stepping Out constitute ‘engines of alternative objectivity’ – alternatives to biomedical knowledge and service provision, in the exploration and cultivation of practices for engaging mental distress and health. The Hearing Voices Movement has cultivated what we are calling an ‘activist science’, which incorporates an ethos of experimentation: engaging, testing, recording and sharing voice-hearing experiences. Their self-help practices can be understood not as about giving space to a reactive victim culture or valorising the subjective perspective of the individual; rather, they are concrete practices and technologies for sharing, recording and substantiating common experience and technical know-how. Stepping Out distinguishes itself from both community theatre and drama therapy through its embracing of high production values, aesthetic standards and critical review processes – components of objective theatrical culture writ-large. While the theatre is associated with affective personal forms of intensity, the experiences of Stepping Out demonstrate the capacities of the theatre troupe to hold open spaces for the encounter of material devices, aesthetic standards and external judgment practices.

These are just two of a wealth of possible examples of ways in which people are working together to generate authority and dignity through experiences and performances of objectivity. Other examples of places where objectivity is generated in participatory mental health practices include clubhouse models, peer-run respites and knowledge-sharing online forums such as *Erowid*. Crucially, these are all spaces and practices for the collectivisation of experience, thus engaging with and generating forms of objectivity. We argue that the political importance of their work to generate alternative objectivity can be articulated in terms of its capacity to overcome alienation and generate dignity. Overcoming alienation both democratises and redistributes power. The practices of Hearing Voices Movement and Stepping Out can be understood as such work of overcoming alienation: enabling subjective lives to create, interact with and transform *shared* forms of life. They do not create merely ‘intersubjective’ spaces that dissolve boundaries between individuals. Both the Hearing Voices Movement and Stepping Out create more than subjective experiences, spaces and forms of life. They practice the complex kind of interdependence embodied in the self-help motto, ‘*You alone can do it but you cannot do it alone*’ (Mowrer, cited in Borkman, 1999). A key issue is the recognition that learning and – more broadly – creative intellectual development are not simply passed on as memory or accomplished explanation from human subject to human subject, but rather are constituted in direct encounters between subjects, drives and objective forms – be that texts, calculations, facts, matter, physical processes, poetry or stories. The craftwork of emancipatory participation is about setting the stage for such encounters, by creating spaces, providing materials, building confidence and so on. It requires holding things open, not prescribing possible pathways of understanding or action. The relationships through which we emerge as collective (and individual) agents include relationships with material forces, objects, bodies and things (Blencowe, 2008). By recognising this, we enrich the politics of mental health, seeing not only ‘alternatives’ and ‘complements’ to the scientific objectivity of the biomedical model but also new modes of objectivity forged in the creative crucibles of collaborative life.

## Notes

1. While Wenger’s (1998) term ‘communities of practice’ is useful for highlighting the potential value of participation in the sharing of projects, our interest here is to focus on one aspect of such practice: the generation of forms of objectivity.
